# Cytochalasin B-Induced Membrane Vesicles from TRAIL-Overexpressing Mesenchymal Stem Cells Induce Extrinsic Pathway of Apoptosis in Breast Cancer Mouse Model

**DOI:** 10.3390/cimb45010038

**Published:** 2023-01-09

**Authors:** Daria S. Chulpanova, Tamara V. Pukhalskaia, Zarema E. Gilazieva, Yuliya V. Filina, Milana N. Mansurova, Albert A. Rizvanov, Valeriya V. Solovyeva

**Affiliations:** Institute of Fundamental Medicine and Biology, Kazan Federal University, 420008 Kazan, Russia

**Keywords:** tumor necrosis factor-related apoptosis-inducing ligand, mesenchymal stem cells, extracellular vesicles, cytochalasin B, extrinsic apoptosis pathway, cancer therapy, breast cancer

## Abstract

Tumor-necrosis-factor-associated apoptosis-inducing ligand (TRAIL) is one of the most promising therapeutic cytokines that selectively induce apoptosis in tumor cells. It is known that membrane vesicles (MVs) can carry the surface markers of parental cells. Therefore, MVs are of interest as a tool for cell-free cancer therapy. In this study, membrane vesicles were isolated from TRAIL-overexpressing mesenchymal stem cells using cytochalasin B treatment (CIMVs). To evaluate the antitumor effect of CIMVs-TRAIL in vivo, a breast cancer mouse model was produced. The animals were intratumorally injected with 50 µg of native CIMVs or CIMVs-TRAIL for 12 days with an interval of two days. Then, tumor growth rate, tumor necrotic area, the expression of the apoptosis-related genes *CASP*8, *BCL-2*, and B*AX* and the level of CASP8 protein were analyzed. A 1.8-fold increase in the *CAS*8 gene mRNA and a 1.7-fold increase in the CASP8 protein level were observed in the tumors injected with CIMVs-TRAIL. The expression of the anti-apoptotic *BCL-2* gene in the CIMV-TRAIL group remained unchanged, while the mRNA level of the pro-apoptotic *BAX* gene was increased by 1.4 times, which indicated apoptosis activation in the tumor tissue. Thus, CIMVs-TRAIL were able to activate the extrinsic apoptosis pathway and induce tumor cell death in the breast cancer mouse model.

## 1. Introduction

The mechanism of conventional tumor therapy approaches, such as chemotherapy and/or radiation therapy, is the activation of the intrinsic or mitochondrial apoptosis pathway in response to cellular stress and DNA damage in the tumor cells [1]. However, both chemotherapy and radiation therapy lack specificity, and, in addition, a large number of tumor cells have mutations in the genes of key participants of the intrinsic apoptosis pathway, which make them resistant to the mitochondrial apoptosis pathway induction [2]. Another apoptosis induction pathway, an extrinsic one, is activated by the selective binding of death receptors (DRs) with the apoptosis-inducing ligand associated with tumor necrosis factor (TRAIL, also known as Apo2L), a member of the tumor necrosis factor (TNF) superfamily. Normally, TRAIL is expressed in the membrane-bound and soluble form by various cells of the immune system. TRAIL has quickly come into the sharp focus of researchers as a promising agent for eradicating tumors due to its ability to induce apoptosis in a wide range of cancer cells without affecting normal tissue [3]. The binding of TRAIL to its death receptors, DR4 and DR5, induces the recruitment of Fas-associated death domain protein (FADD). FADD connects to procaspases 8 and 10 to form a death-inducing signaling complex (DISC). DISC additionally activates caspase 8 (CASP8), which directly actuates other effector caspases which facilitate the final stages of apoptosis [4].

The promising results of preclinical studies have led to an initiation of a large number of clinical trials investigating the therapeutic efficacy of recombinant human TRAIL (rhTRAIL) or monoclonal antibodies (mAbs) against TRAIL receptors (TRAILRs) in cancer patients as monotherapy or in combination with chemotherapy [5,6,7]. The therapy was shown to be safe and well tolerated by patients, but the therapeutic results were not as significant as it was expected [8,9]. For example, one of the most effective drugs among those investigated was mAb Mapatumumab, which caused two partial and one complete response in 40 patients with non-Hodgkin’s lymphoma [10]. No significant therapeutic activity has also been shown for other mAbs [11]. The observed low efficiency of apoptosis induction in patients could be associated with a number of factors, such as a short half-life of rhTRAIL, weak mAb-mediated induction of apoptosis due to the formation of TRAILR dimers, and congenital or acquired resistance to the activation of TRAIL-mediated apoptosis [12].

In order to overcome disadvantages, such as the short half-life of rhTRAIL and the inability of mAbs to cross-link TRAIL receptors that require trimerization, the cell-mediated delivery approach is being extensively explored. One of the most promising targeted delivery cell types is mesenchymal stem cells (MSCs), which can selectively migrate and deliver therapeutic agents toward tumor regions [13]. A number of studies have shown that genetically modified MSCs overexpressing TRAIL were able to induce apoptosis in various tumors in vivo [14], including TRAIL-resistant ones, such as Ewing sarcoma [15]. Despite the encouraging results, there are a number of limitations to the inclusion of genetically modified MSCs in clinical practice, as they are associated with the possibility of their undesirable malignant transformation and the ability to support tumor growth and development [16].

One option to overcome the difficulties of cell therapy is the use of extracellular vesicles (EVs) isolated from genetically modified cells. EVs are capable of carrying biologically active molecules of parental cells and, therefore, can be used as a delivery vehicle for antitumor therapeutic agents [17,18]. The use of EVs, isolated from MSCs-TRAIL, inhibited tumor progression in an in vivo model [19,20] and led to partially overcoming resistance in insensitive cells in vitro [21].

In this study, cytochalasin B-induced membrane vesicles (CIMVs-TRAIL) were isolated from genetically modified MSCs-TRAIL and their ability to induce TRAIL-mediated apoptosis in vitro and in vivo in breast cancer mouse models was analyzed.

## 2. Materials and Methods

### 2.1. Cells and Culture Conditions

The adipose tissue samples were obtained from a healthy female donor that underwent abdominal liposuction at the Republican Clinical Hospital in accordance with approved ethical standards and current legislation (the protocol was approved by the Committee on Biomedical Ethics of Kazan Federal University (No. 3, 03/23/2017)). Informed consent was obtained from the donor.

Briefly, the adipose tissue samples were incubated at 37 °C for 60 min in PBS containing 2 mg/mL of collagenase (Rosmedbio, Saint-Petersburg, Russia) and shacked (120 cycles/min) for 60 min. After that tissue was centrifuged at 1400× rpm for 5 min, the stromal vascular fraction was collected, washed, and resuspended in a DMEM/F12 medium (PanEco, Moscow, Russia) supplemented with 10% fetal bovine serum (FBS, Invitrogen, USA), 2 mM of L-glutamine and antibiotics (100 U/mL of penicillin, 100 μg/mL of streptomycin, PanEco, Russia) and incubated at 37 °C with a 5% CO_2_ density in air and relative humidity of 95%. The MSCs were used in passages 6–8.

Human estrogen receptor (ER)-positive MCF-7 breast cancer cells (#HTB-22) and 293T embryonic kidney cells (#CRL-3216) were obtained from the American Type Culture Collection (ATCC, Manassas, VA, USA). All of the cells were cultured in a DMEM medium (PanEco, Moscow, Russia) with 4500 mg/L of glucose, 10% FBS (HyClone, Logan, UT, USA), 4 mM of L-glutamine, and antibiotics (PanEco, Moscow, Russia) and were incubated at 37  °C, 5% CO_2_/95% humidified air. Cell morphology analysis was carried out using an Axio Observer.Z1 (CarlZeiss, Jena, Germany) microscope and Axio Vision Rel. 4.8 software.

### 2.2. Lentivirus Production

In order to produce lentiviral particles, vector plasmids pLX303-TRAIL and pLX303-BFP were produced by sub-cloning the *TRAIL* gene or *BFP* gene from the donor vector pDONR221-TNFSF10 (HsCD0042890, Harvard Plasmid Database, Boston, MA, USA) or pDONR223-BFP (#25891, Addgene, Watertown, MA, USA), respectively, into the lentiviral plasmid vector pLX303 (#25897, Addgene, Watertown, MA, USA) by LR recombination using the Gateway™ LR Clonase™ II Enzyme mix (#11791020, Invitrogen, Waltham, MA, USA) according to the manufacturer’s recommendations.

For LV production, the 293T cells were plated at a concentration of 2.5 × 10^6^ cells in 10 cm Petri dishes and incubated for 24 h. After that, the cells were transfected with an expression plasmid (pLX303-TRAIL or pLX303-BFP), pCMV-dR8.74 packaging plasmid (#22036, Addgene, Watertown, MA, USA), and a pCMV-VSV g enveloping plasmid (#8454, Addgene, Watertown, MA, USA) using polyethyenimine, where the ratio of μg DNA:μg PEI was 1:3. The viral particles were harvested at 48, 72, and 96 h post-transfection and concentrated using ultracentrifugation for 2 h at 26,000 rpm at 4 °C.

### 2.3. Genetic Modification and Selection

The MSCs were seeded on a 6-well plate at a concentration of 5 × 10^4^ cells and incubated for 24 h. After that, the LV-TRAIL or LV-BFP were diluted in serum-free Opti-MEM medium (#31985062, Gibco, Waltham, MA, USA) and added to the cells at a multiplicity of infection (MOI) of 10. The medium with viruses was completely replaced with the fresh medium 6 h after transduction. Genetically modified cells were selected with blasticidin S (5 μg/mL, Invitrogen, Waltham, MA, USA) for 10 days.

### 2.4. Quantitative Polymerase Chain Reaction (qPCR)

To confirm the genetic modification, the total RNA was extracted from the native MSCs, MSCs-BFP, and MSCs-TRAIL using the TRIzol reagent (Invitrogen, USA) according to the manufacturer’s instructions. Reverse transcription was conducted using a RevertAid RT Reverse Transcription Kit (K1691, Thermo Scientific, Waltham, MA, USA).

For the *18S rRNA* and *TRAIL* gene cDNAs, the PCR primers and probe were designed using the GenScript Online Real-time PCR (TaqMan) Primer Design Tool (GenScript, Piscataway, NJ, USA) and synthesized by Evrogen, Russia (Table 1). PCR was performed in a reaction mixture consisting of 2 μL of 5 × buffer (Evrogen, Moscow, Russia), 0.3 μL of primers and a probe mix (final primer concentration of 300 nM each), 4.7 μL of sterile H_2_O (Evrogen, Moscow, Russia), and 1 μL of the cDNA template. The thermal conditions were carried out in a CFX96 Touch™ Real-Time PCR Detection System (BioRad Laboratories, Hercules, CA, USA) as follows: one cycle of denaturation at 95 °C for 3 min followed by 45 cycles of amplification at 95 °C for 10 s and 55 °C for 30 s. The *TRAIL* gene expression level was normalized to the *18S rRNA* level. Relative quantification was performed using the comparative threshold cycle (ΔΔCT) method.

### 2.5. Western Blot Analysis

To confirm the presence of TRAIL protein in the native and genetically modified MSCs, 1 × 10^6^ cells were lysed in a RIPA buffer containing a Halt™ Protease and Phosphatase Inhibitor Cocktail (#78444, Thermo Scientific, Waltham, MA, USA). The total protein concentration in the isolated samples was determined using a Pierce BCA Protein Assay Kit (#23225, Thermo Scientific, Waltham, MA, USA) according to the manufacturer’s recommended protocol. Equal volumes of total protein (30 µg) were used to denature the protein via electrophoresis in 4-12% SDS-PAGE gradient. The resolved proteins were transferred onto a PVDF membrane (#88520, BioRad, Hercules, CA, USA) with the following incubation in the blocking buffer (5% non-fat milk). The membrane was incubated with the primary rabbit anti-TRAIL antibodies (0.2 µg/mL, #9959, Abcam, Cambridge, UK) overnight at 4 °C. Excessive primary antibodies were removed by washing. After that, the membranes were incubated with the secondary antibody, horseradish peroxidase (HRP)-conjugated goat anti-rabbit immunoglobulin G (1:2000; #8a0467j, American Qualex Antibodies, Orange, CA, USA) or with anti-β-actin HRP-conjugated antibody (1:1000; #A00730, GenScript, Piscataway, NJ, USA) for 2 h at room temperature. The membrane was washed, exposed to clarity enhanced chemiluminescence (ECL) reagent (#1705061, Bio-Rad, Hercules, CA, USA) for 3 min at room temperature, and visualized using a ChemiDoc XRS^+^ system (BioRad, Hercules, CA, USA).

### 2.6. Confirmation of TRAIL Protein Membrane Localization

In order to confirm the presence of TRAIL on the membrane of the genetically modified MSCs 1 × 10^5^ native MSCs, MSCs-TRAIL and MSCs-BFP were detached from the culture flask using Accutase™ cell dissociation reagent (#A1110501, Gibco, Waltham, MA, USA) and washed once with PBS. The washed cells were stained with anti-human CD253 (TRAIL) antibody (#308210, BioLegend, San Diego, CA, USA) for 30 min at room temperature in the dark. The stained cells were washed with PBS and analyzed by flow cytometry using FACSAria III (BD Biosciences, San Jose, CA, USA).

### 2.7. Immunophenotyping of MSCs

The native and genetically modified MSCs were assessed for surface marker expression via flow cytometry. The cells were trypsinized, washed with PBS, and stained with fluorochrome-conjugated anti-human antibodies against CD29, CD44, CD73, CD90, and CD105, as well as a negative cocktail (CD34, CD11b, CD19, CD45, and HLA-DR) (all from BD Stemflow™ Human MSC Analysis Kit, BD Biosciences, San Jose, CA, USA) for 30 min in the dark at room temperature. The cells were washed once with PBS and analyzed by flow cytometry using FACSAria III (BD Biosciences, San Jose, CA, USA), and the data were analyzed using BD FACSDiva™ software version 7.0.

### 2.8. Cell Viability Assay

To determine the number of alive, apoptotic, and necrotic cells 1 × 10^5^ native MSCs, MSCs-TRAIL and MSCs-BFP were seeded in 6-well plates in DMEM/F12. After 24 h, the cells were trypsynized, washed twice with PBS, and stained with APC Annexin V Apoptosis Detection Kit with PI (#40932, BioLegend, San Diego, CA, USA) according to the manufacturer’s protocol. The stained cells were analyzed by flow cytometry using the FACSAria III (BD Biosciences, San Jose, CA, USA).

### 2.9. Cell Proliferation Assay

To determine the effect of genetic modification on the proliferation of MSCs, the native and genetically modified MSCs were seeded on a 96-well plate at a concentration of 5 × 10^3^ cell/well and incubated for 24 h. Then, MTS (#ab223881, Abcam, Cambridge, UK) and PMS (#P9625, Sigma-Aldrich, Saint Louis, MO, USA) reagents were mixed in a ratio of 20:1, and 10 µL of the mixture was added to the cells. After the incubation for 30 min at 37 °C, the absorbance (490 nm) in the wells was measured using an Infinite M200Pro (Tecan Trading AG, Maennedorf, Switzerland) with reference wavelength = 630 nm.

### 2.10. Analysis of the Cytokine Profile in the Conditioned Medium

To analyze changes in the cytokine/chemokine profile of the MSCs after the genetically modified native MSCs, MSCs-BFP, and MSCs-TRAIL were seeded at a density of 2 × 10^5^ cells in T75 culture flasks. The conditioned medium was harvested after 24, 48, and 72 h of cultivation, centrifuged (1500 rpm for 5 min at room temperature), and stored at −80 °C. The Human Chemokine 40-plex Panel (#171ak99mr2, BioRad Laboratories, USA) was used to analyze the conditioned medium samples according to the manufacturer’s recommendations. Human Chemokine 40-plex Panel detects C-C motif chemokine ligand 21 (CCL21), C-X-C motif chemokine ligand 13 (CXCL13), CCL27, CXCL5, CCL11, CCL24, CCL26, CX3CL1, CXCL6, granulocyte-macrophage colony-stimulating factor (GM-CSF), CXCL1, CXCL2, CCL1, interferon ϒ (IFN-ϒ), interleukin 1β (IL1β), IL2, IL4, IL6, IL8/CXCL8, IL10, IL16, IP10/CXCL10, I-TAC/CXCL11, monocyte chemoattractant protein-1 (MCP-1)/CCL2, MCP-2/CCL8, MCP-3/CCL7, MCP-4/CCL13, macrophage-derived chemokine (MDC)/CCL22, macrophage migration inhibitory factor (MIF), CXCL9, macrophage inflammatory protein 1α (MIP-1α)/CCL3, MIP-1δ/CCL15, MIP-3α/CCL20, MIP-3β/CCL19, myeloid progenitor inhibitory factor 1 (MPIF-1)/CCL23, CXCL16, stromal cell-derived factor 1α+β (SDF-1α+β)/CXCL12, CCL17, CCL25, and tumor necrosis factor α (TNF-α). Fifty microliters of each sample were used to determine the cytokine concentration, and the collected data were analyzed using a Luminex 200 analyzer with MasterPlex CT control and QT analysis software (MiraiBio division of Hitachi Software, San Francisco, CA, USA).

### 2.11. Isolation of Cytochalasin B-Induced Membrane Vesicles

To isolate the membrane vesicles from the native and genetically modified MSCs, the cytochalasin B drug was used. For this, 1 × 10^6^ native MSCs, MSCs-BFP, or MSCs-TRAIL was washed in PBS and incubated in DMEM without serum with the 10 mg/mL cytochalasin B from Drechslera dematioidea (#C6762-5MG, Sigma-Aldrich, Saint Louis, MO, USA) for 30 min at 37 °C, stirring every 10 min. Next, to mechanically affect the membrane of the cells, the suspension was vortexed for 60 s. After that, the cells were centrifuged for 10 min at 500 rpm at room temperature, and the resulting supernatant was transferred to a new centrifuge tube. In the next step, the suspension with the vesicles was centrifuged for 10 min at 700 rpm, and the resulting supernatant was transferred to a new centrifuge tube. After the final centrifugation step (15 min at 12,000 rpm), the pelleted vesicles were resuspended in a culture medium, PBS, or another buffer, depending on the purpose of the further experiment. The concentration of the total protein in the isolated CIMVs was determined using a Pierce BCA Protein Assay Kit (Thermo Scientific, Waltham, MA, USA) according to the manufacturer’s recommended method.

### 2.12. Assessment of TRAIL mRNA and Protein Inside and on the Surface of the CIMVs

In order to evaluate the presence of the *TRAIL* gene mRNA in the CIMVs isolated from native MSCs (native CIMVs), MSCs-BFP (CIMVs-BFP), and MSCs-TRAIL (CIMVs-TRAIL) the tRNA was extracted from the newly isolated CIMVs, and qPCR was performed as described in Section 2.4.

In order to evaluate the presence of the TRAIL protein, the native CIMVs, CIMVs-BFP, and CIMVs-TRAIL were isolated, as previously described, and lysed in an RIPA buffer containing a Halt™ Protease and Phosphatase Inhibitor Cocktail (Thermo Scientific, Waltham, MA, USA). Then, 50 µg of each CIMV was used for the following Western blot analysis, which was performed as described in Section 2.5.

In order to analyze the localization of the TRAIL protein, the native CIMVs, CIMVs-BFP, and CIMVs-TRAIL were isolated, as previously described, and stained with anti-human CD253 (TRAIL) antibody (#308210, BioLegend, San Diego, CA, USA) for 30 min at room temperature in the dark. The staining was assessed using the FACSAria III flow cytometer (BD Biosciences, San Jose, CA, USA).

### 2.13. Analysis of Typical Extracellular Vesicle Markers in CIMVs

In order to determine the typical EV markers in the resulting CIMVs, they were isolated from 1 × 10^6^ native MSCs, MSCs-BFP, and MSCs-TRAIL, washed once with PBS and stained with 1 µL of anti-CD63 (PerCP/Cy5.5) (#353020, BioLegend, San Diego, CA, USA), anti-CD81 (PE/Cy7) (#349512, BioLegend, San Diego, CA, USA), and anti-tumor susceptibility 101 (TSG101) (PE) (#ab209927, Abcam, Cambridge, UK) antibodies against specific surface markers for 30 min in the dark at room temperature. After that, the CIMVs were washed twice with PBS and analyzed using flow cytometer FACSAria III (BD Biosciences, San Jose, CA, USA) and BD FACSDiva™ software version 7.0. To analyze the presence of the intracellular markers, heat-shock protein 70 kDa (Hsp70) and calnexin, the newly isolated CIMVs were fixed with 0.01% formaldehyde for 15 min at RT and washed with PBS for 5 min. Next, the CIMV membranes were permeabilized using 0.1% Triton X-100 for 15 min at room temperature. After that, the samples were washed twice with PBS and stained with anti-Hsp70 (FITC) (#648004, Biolegend, San Diego, CA, USA) and anti-Calnexin (Alexa Fluor 594) (#ab203439, Abcam, Cambridge, UK) antibodies for 30 min in the dark at room temperature. The stained vesicles were washed twice with PBS and analyzed using FACSAria III (BD Biosciences, San Jose, CA, USA) and BD FACSDiva™ software version 7.0.

### 2.14. Determination of CIMV Size

The CIMVs were isolated from native MSCs, MSCs-BFP, and MSCs-TRAIL, as previously described, and analyzed using flow cytometer BD FACSAria III (BD Biosciences, San Jose, CA, USA). The size of the vesicles was determined comparatively to a mixture of calibration particles (0.22–0.45–0.88–1.34 μm) (Spherotech, Lake Forest, IL, USA). In all of the experiments with the CIMVs, a violet laser (Ex = 405, Em = 450) was used to detect particles from 200 nm in diameter. For sizing the CIMVs by scanning electron microscopy (SEM), the CIMVs were isolated as previously described, resuspended in PBS and applied on glass slides by centrifugation at 3000× rpm for 30 min at room temperature. The CIMVs were fixed with 10% formalin for 15 min, dehydrated through an ethanol gradient from 30% to absolute and air-dried for 24 h. Prior to imaging, the samples were coated with gold/palladium in a Quorum T150ES sputter coater (Quorum Technologies Ltd., Laughton, UK) and viewed for analysis by an SEM Merlin (Carl Zeiss, Jena, Germany). Two biological replicates were completed for the experiment.

### 2.15. In Vitro Assessment of CIMV-TRAIL Anti-Cancer Activity

To analyze the induction of CIMV-TRAIL-mediated apoptosis in tumor cells, 5 × 10^4^ MCF-7 cells were cultured in a 12-well plate for 24 h. Then, the native CIMVs, CIMVs-BFP, or CIMVs-TRAIL (50 mg/mL) were added to the cells and incubated for 24 h. Then, the cells were trypsinized, washed with PBS, and resuspended in Cell Staining Buffer (#420201, BioLegend, San Diego, CA, USA). The number of apoptotic and necrotic cells was determined using the APC Annexin V Apoptosis Detection Kit with PI (#3804660, BioLegend, San Diego, CA, USA). The cells were stained according to the protocol recommended by the manufacturer in the dark for 30 min at room temperature and analyzed on a FACSAria III flow cytometer (BD Biosciences, San Jose, CA, USA). The number of cells in which caspase 8 was activated was determined using Vybrant^TM^ FAM^TM^ Caspase-8 Assay Kit (#V35119, Invitrogen, Waltham, MA, USA). Briefly, the cells were stained with the FLICA^TM^ working solution for 1 h at 37 °C and 5% CO_2_, protected from light. After that, the cells were washed twice with PBS, stained with propidium iodide for 10 min and analyzed on a FACSAria III flow cytometer (BD Biosciences, San Jose, CA, USA).

### 2.16. Analysis of Changes in the Expression of Apoptotic Regulator Genes in Tumor Cells after Interaction with CIMVs

After the analysis of apoptosis/necrosis and caspase 8 activation, the MCF-7 tumor cells were washed twice with PBS, and the total RNA was isolated using the TRIzol reagent (Invitrogen, Waltham, MA, USA). The synthesis of cDNA by reverse transcription and real-time PCR analyzing the expression of key apoptotic regulator genes *BCL-2*, *BAX*, and *CASP8* was carried out in accordance with the procedure described earlier in Section 2.4. Primers and fluorescent probes specific for cDNA of *18S rRNA*, *CASP8*, *BCL-2*, and *BAX* genes were developed using the GenScript Online Real-time PCR (TaqMan) Primer Design Tool (GenScript, Piscataway, NJ, USA) software and synthesized by Evrogen (Moscow, Russia) (Table 1).

### 2.17. Animals

In order to analyze the antitumor activity of CIMVs in vivo, a breast cancer mouse model was produced. For this, female Balb/c nude mice (4 weeks old) were purchased from the vivarium of laboratory animals of the Institute of Bioorganic Chemistry named after Academicians M. M. Shemyakin and Yu. A. Ovchinnikov (Pushchino, Russia). The animals were housed in pathogen-free conditions with filtered air, and autoclaved food and water were available ad libitum. All of the experiments were carried out in compliance with the procedure protocols approved by the Kazan Federal University local ethics committee (protocol No. 33, date 25 November 2021) according to the rules adopted by Kazan Federal University and Russian Federation Laws.

### 2.18. Creation of Breast Cancer Mouse Tumor Model

One week before the administration of the tumor cells, the mice were subcutaneously injected with β-estradiol (350 µg/kg, #436320250, Acros Organics, Geel, Belgium) in 100 µL of sesame oil in order to provide appropriate tumor growth estrogen levels in the animals. Then, 1 × 10^7^ MCF-7 cells were injected into each mouse in the volume of 100 µL suspension of PBS and Matrigel (#356235, BD Biosciences, San Jose, CA, USA) (1:1), subcutaneously in the right flank with an insulin syringe. The tumors were measured every 3–4 days with a Vernier caliper, and the tumor volume (TV) was calculated as TV (mm^3^) = d^2^ × D/2, where d and D are the shortest and the longest diameter, respectively.

### 2.19. Treatment of Mice with CIMVs

The mice were randomized into three groups with 5 animals per group: (1) control animals injected with PBS; (2) animals injected with native CIMVs; (3) animals injected with CIMVs-TRAIL. The treatment was started at 100 mm^3^ tumor volume (TV). The treatment schedule was composed of 5 intertumoral injections every 48 h, with 50 μg of CIMVs in 20 µL of PBS per injection. Then, 24 h after the last treatment, the mice were euthanized in compliance with the protocols approved by the Kazan Federal University local ethics committee (protocol No. 33, date 25 November 2021). The tumors were collected and used for the subsequent analysis of antitumor activity.

### 2.20. Histological Analysis of the Tumors after CIMV Administration

The xenografts were harvested after sacrificing the mice, fixed in 10% buffered formalin for 48 h, and stored in 30% saccharose. The fixed tumor tissue was frozen in a tissue-freezing medium (Tissue-Tek O.C.T. Compound, Sakura, Torrance, CA, USA). Then, transverse sections of 5–7 µm were cut with a Microm HM 560 cryostat (Thermo Scientific, Waltham, MA, USA). The histological analysis was processed with conventional hematoxylin and eosin (H&E) staining for the visualization of general tissue morphology using a whole slide scanner NanoZoomer S60 (Hamamatsu Photonics, Hamamatsu, Japan) with 40× magnification. Five sections for each tumor were randomly assessed during the statistical analysis of the percentage of necrotic area in the tumor tissue. The area of necrosis within the whole-tumor section was determined visually, and the proportion of necrotic tumor over the whole section was calculated using ImageJ (Fiji) software.

### 2.21. Analysis of Induction of Apoptosis in Tumor Cells after CIMV Administration

In order to analyze the induction of apoptosis in tumor xenografts, the total RNA was isolated from the tumor tissue for subsequent analysis. For this, the tumor tissue in 200 µL of TRIzol was homogenized with 0.5–1 mm glass beads (#Z250465 and Z250473, Sigma-Aldrich, Saint Louis, MO, USA) using a FastPrep-24™ homogenizer (MP Biomedicals, Eschwege, Germany). After that, the total RNA was isolated, and the cDNA was synthesized. The expression of the apoptotic genes *CASP8*, *BCL-2*, and *BAX* in the tumor tissue was determined using qPCR, as described in Section 2.4.

The total protein was isolated from the tumor tissue via homogenization with 0.5–1 mm glass beads in an RIPA buffer containing a Halt™ Protease and Phosphatase Inhibitor Cocktail (#78444, Thermo Scientific, Waltham, MA, USA). The total protein concentration in the isolated samples was determined using a Pierce BCA Protein Assay Kit (#23225, Thermo Scientific, Waltham, MA, USA) according to the manufacturer’s recommended method. Equal volumes of total protein (30 µg) were used to analyze the activated caspase 8 protein level. Western blot analysis was performed, as described in Section 2.5, using primary antibody against cleaved caspase 8 in a dilution of 1:1000 (#9496, Cell Signaling Technology, Danvers, MA, USA). The caspase 8 bands were normalized to β-actin, and densitometric analysis was performed using ImageLab software version 6.0.1 (BioRad Laboratories, Hercules, CA, USA).

### 2.22. Statistical Analysis

Statistical analysis was undertaken using GraphPad Prism 7 software (GraphPad Software, San Diego, CA, USA), one-way ANOVA followed by Tukey HSD post hoc comparisons test. The significant probability values are denoted as * *p* < 0.05, ** *p* < 0.01, and *** *p* < 0.001, **** *p* < 0.0001. The data were represented as the mean of replicates with their corresponding standard deviation (SD).

## 3. Results

### 3.1. Genetically Modified MSCs-TRAIL Remain Properties of Stem Cells

**Figure 1:**
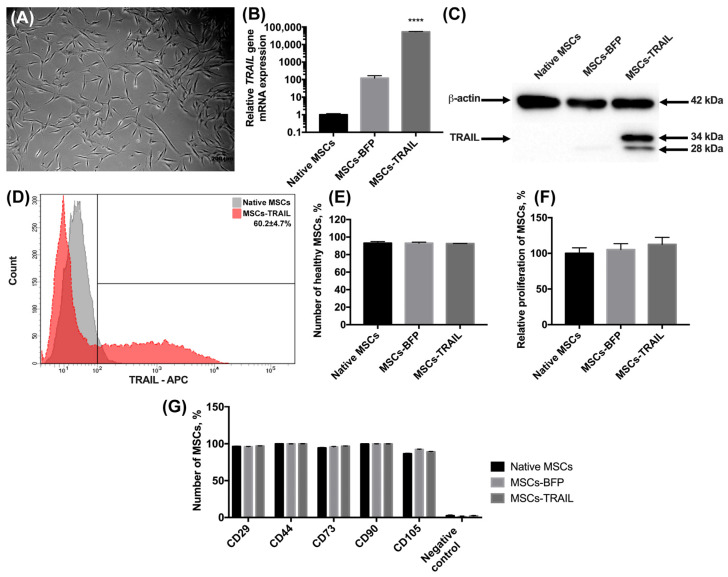
Characterization of MSCs after genetic modification. (**A**) Newly isolated MSCs. Phase-contrast light microscopy, scale bar: 200 µm. (**B**) Genetically modified MSCs-TRAIL and MSCs-BFP expressed by 52,591 times and 100 times more copies of *TRAIL* gene, respectively, compared to native MSCs. *18S RNA* reference gene has been used for normalization of the data. Bars represent the mean of three replicates with their corresponding standard deviation (*n* = 3). (**C**) Expression of two isoforms of TRAIL protein in MSCs-TRAIL (28 and 34 kDa) was confirmed using Western blot. (**D**) About 60% of MSCs-TRAIL carried the TRAIL on their surface. (**E**) Number of alive MSCs was the same in all the groups after 48 h of cultivation. Bars represent the mean ± SD (*n* = 3). (**F**) TRAIL overexpression also failed to affect relative proliferation rate of MSCs. Each value is presented as the % in relation to the control (native MSCs) group. Bars represent the mean ± SD (*n* = 3). (**G**) The immunophenotype of native and genetically modified MSCs also remained unchanged after genetic modification. Data were received by flow cytometry and are shown as the mean percentage ± SD (*n* = 3). **** *p*  <  0.0001.

The MSCs were isolated from human adipose tissue, as described in the Materials and methods section. The morphology of the isolated cells typically appeared to be fibroblast-like and spindle-shaped (Figure 1A). The cells expressed typical MSC markers CD29, CD44, CD73, CD90, and CD105 and did not express hematopoietic stem cell markers CD34, CD11b, CD19, CD45, and HLA-DR. Further, the MSCs were genetically modified with the lentiviruses-encoding *TRAIL* (MSCs-TRAIL) or *BFP* genes (MSCs-BFP)*,* and the genetically modified cells were selected as described in the Materials and Methods section. The mRNA level of the *TRAIL* gene had a 52,591-fold increase (*n* = 3, **** *p* < 0.0001) in MSCs-TRAIL compared to the native MSCs (Figure 1B). It is also worth noting that the *TRAIL* gene mRNA expression also had a 100-fold increase in MSCs-BFP (Figure 1B). The protein expression in MSCs-TRAIL was confirmed by Western blot analysis. Two TRAIL isoforms of 28 and 34 kDa were detected (Figure 1C). To determine the subcellular location of the TRAIL protein, which should be located on the membrane to effectively induce apoptosis in cancer cells, the native and genetically modified MSCs were stained with fluorescent antibodies to CD253 (TRAIL). It was shown that 60.2 ± 4.7% of MSCs-TRAIL carried the TRAIL on their surface (Figure 1D).

We have also analyzed the effect of genetic modification on the viability and proliferation rate of MSCs. There was no statistically significant difference in the number of alive native MSCs (93.15% ± 1.8%), MSCs-BFP (93.06% ± 1.21%), and MSCs-TRAIL (92.6% ± 0.14%) (Figure 1E) 48 h after cultivation. The proliferation rate of the genetically modified MSCs-BFP (105.3% ± 8.2%) and MSCs-TRAIL (112.6% ± 9.87%) also remained unchanged in comparison with the native MSCs (100% ± 7.7%) (Figure 1F).

Flow cytometry analysis of the surface antigens showed that both MSCs-BFP and MSCs-TRAIL retained the typical markers of mesenchymal stem cells. It was shown that the expression of CD90 on the surface of the native MSCs was 99.6% ± 0.1%, CD105—86.5% ± 0.7%, CD73—94.4% ± 0.6%, CD44—99.8% ± 0.1%, and CD29—96.3% ± 0.5%. The expression of CD90, CD105, CD73, CD44, and CD29 was observed on the surface of 99.7% ± 0.1%, 92.2% ± 0.5%, 95.7% ± 0.6%, 99.8% ± 0.1%, 95.9% ± 0.4% MSCs-BFP, respectively. MSCs-TRAIL expressed CD90, CD105, CD73, CD44, and CD29 on the surface of 99.7% ± 0.1%, 89.1% ± 0.4%, 96.7% ± 0.3%, 99.9% ± 0.1%, and 96.9% ± 0.3% cells, respectively. At the same time, both native and genetically modified MSCs were negative for hematopoietic stem cell markers CD34, CD11b, CD19, CD45, and HLA-DR (Figure 1G and Figure S1).

**Figure 2:**
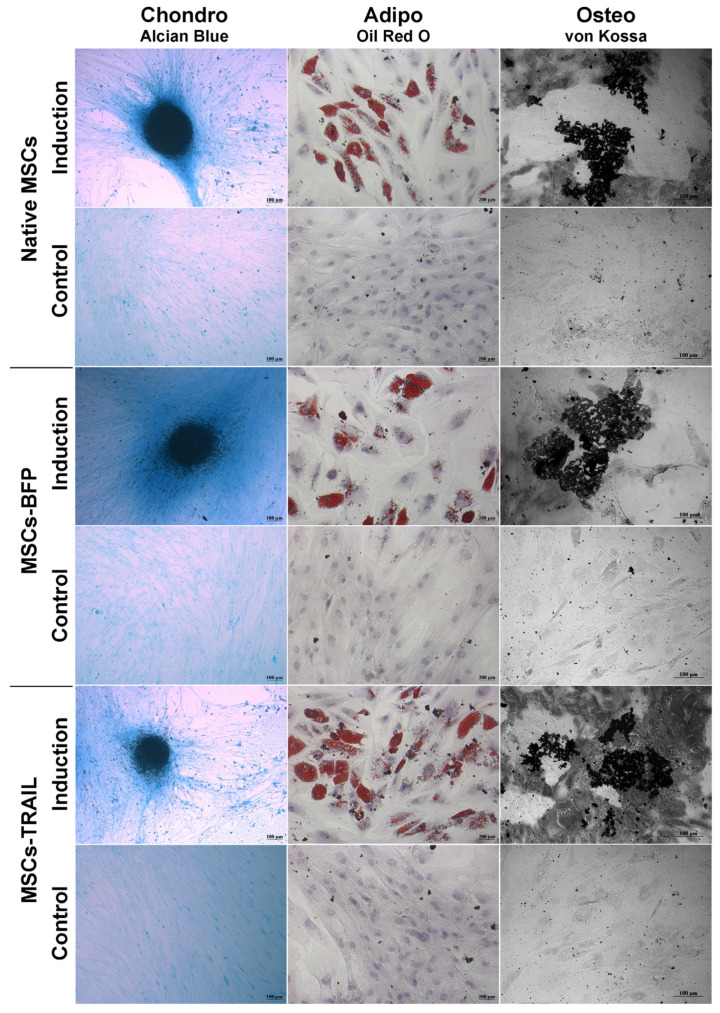
Osteogenic, adipogenic and chondrogenic differentiation of hADSCs. Phase contrast microscope images. To differentiate toward adipogenic lineage, native and TRAIL-genetically modified cells were cultured in reprogramming medium for 14 days. At day 14, cells were fixed and stained with Oil Red O. For osteogenic differentiation, the cells were cultured in reprogramming medium for 28 days. At day 28, cells were fixed and analyzed by von Kossa staining. Chondrogenic differentiation was determined by staining with Alcian blue on day 21 after seeding (adipogenic: scale bar, 200 µm; chondrogenic and osteogenic differentiation: scale bar, 100 µm).

Genetically modified MSCs-BFP and MSCs-TRAIL, similar to native MSCs, hold the ability to differentiate into osteogenic, chondrogenic, and adipogenic directions (Figure 2).

### 3.2. TRAIL Overexpression Results in the Significant Decrease of Pro-Inflammatory Cytokine Secretion

The levels of pro-inflammatory cytokines and chemokines were analyzed in a conditioned medium of native MSCs, MSCs-BFP, and MSCs-TRAIL (Table 2). A strong association between TRAIL overexpression and a decrease in the level of several cytokines was observed. The levels of cytokines CCL21, eotaxin, CXCL1, CXCL2, CCL7, CCL19, and CCL25, which are capable of supporting tumor invasion, were reduced in CM from MSCs-TRAIL by 1.2, 1.3, 4-8, 3.4-1.3, 1.5, and 1.3 times, respectively (*n* = 3, ** *p* < 0.01).

The expression of immunomodulating cytokines IL10, CCL15, CCL17, and CXCL5 was also 1.6-, 2.2-, 1.3-, and 2-fold decreased in the conditioned medium of MSCs-TRAIL compared to the CM of the native MSCs (*n* = 3, ** *p* < 0.01).

The most significant changes were observed in the level of pro-inflammatory cytokines IL1β, IL6, and IL8, the secretion of which was reduced by 2, 4.4, and 5.3 in the MSCs-TRAIL CM sample compared with the native MSCs over a 24 h period. By 72 h, the level of suppression decreased yet remained significant. The secretion of IL1β, IL6, and IL8 was reduced in MSCs-TRAIL by 2.3, 2, and 3.2 times, respectively (*n* = 3, *** *p* < 0.001).

### 3.3. CIMVs Isolated from MSCs-TRAIL Carry TRAIL Protein

**Figure 3:**
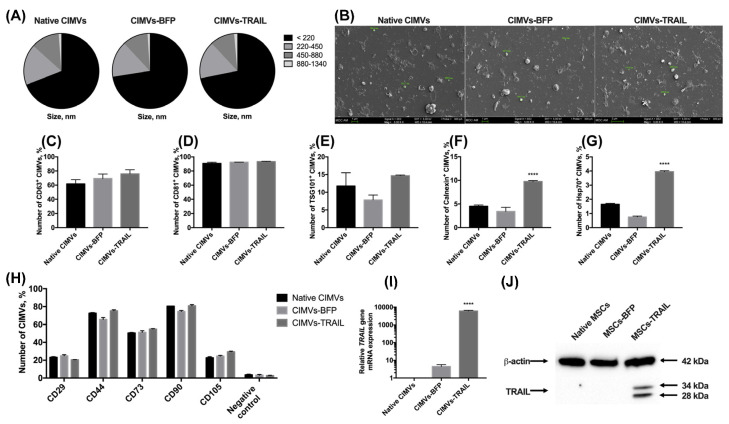
The size of cytochalasin B-induced membrane vesicles (CIMVs) was analyzed using flow cytometry (**A**) and SEM (**B**), where the majority of the CIMVs were less than 220 nm in diameter. The isolated vesicles expressed the major markers of extracellular vesicles CD63 (**C**) and CD81 (**D**). At the same time, the expression level of TSG101 (**E**) and intracellular proteins calnexin (**F**) and Hsp70 (**G**) was low. (**H**) The expression of typical surface MSC markers was significantly reduced in both native and genetically modified CIMVs compared to the parental cells. The presence of the *TRAIL* gene mRNA (**I**) and TRAIL protein (**J**) in the isolated CIMVs was confirmed using qPCR and Western blot, accordingly. Each box represents the mean ± SD (*n* = 3) of three replicates. **** *p*  <  0.0001.

After the genetic modification and analysis of the properties of MSCs, membrane vesicles were isolated from them using a protocol based on the treatment with cytochalasin B. The size of the isolated vesicles was analyzed using flow cytometry. It was shown that the majority (66.9 ± 3.8%) of the native CIMVs was 220 nm or below; the rest of the population consisted of vesicles ranging in size from 220 to 450 nm (17.0 ± 1.8%), from 450 to 880 nm (11.9 ± 1.9%), and from 880 nm to 1340 nm (1.1 ± 0.2%). The size of the CIMVs-BFP did not differ significantly (220 nm or below—72.5 ± 2.7%; 220–450 nm—15.5 ± 0.4%; 450–880 nm—11.0 ± 1.9%; 880–1340 nm—1.0 ± 0.4%). A similar size distribution of vesicles in the population was also found for CIMVs-TRAIL (220 nm or below—71.8 ± 3.4%; 220–450 nm—16.2 ± 1.4%; 450–880 nm—10.6 ± 2.0%; ± 0.1%) (Figure 3A). The size of the CIMVs was also analyzed using SEM and corresponded to the data received by flow cytometry. CIMVs had a typical EVs spherical shape and presented uneven non-smooth surfaces (Figure 3B).

We also performed a minimal analysis of surface and intracellular proteins typical for exogeneous extracellular vesicles according to MISEV2018 [22]. The isolated native CIMVs, CIMVs-BFP and CIMVs-TRAIL expressed the main markers of extracellular vesicles. Namely, CD63 was detected on the surface of 61.7 ± 6.0% native CIMVs, 69.3 ± 6.4% of CIMVs-BFP and 75.9 ± 5.7% of CIMVs-TRAIL (Figure 3C). The number of CD81^+^ native CIMVs, CIMVs-BFP and CIMVs-TRAIL was 90.8 ± 1.6%, 92.4 ± 0.1% and 93.0 ± 0.4%, respectively (Figure 3D). The TSG101 protein was found on the surface of 11.7 ± 3.7% of native CIMVs, 7.8 ± 1.4% of CIMVs-BFP and 14.6 ± 0.2% of CIMVs-TRAIL (Figure 3E). The endoplasmic reticulum (ER) protein calnexin was hardly detected inside native CIMVs (4.5 ± 0.2%) and CIMVs-BFP (3.4 ± 0.9%) (Figure 3F). However, the level of calnexin in CIMVs-TRAIL was significantly higher (9.7 ± 0.2%, *n* = 3, **** *p* < 0.0001). Hsp70 was almost undetectable (native CIMVs, 1.6 ± 0.1%, CIMVs-BFP, 0.7 ± 0.1%, CIMVs-TRAIL, 3.9 ± 0.1%) (Figure 3G).

Since CIMVs were isolated from mesenchymal stem cells, we also analyzed the presence of typical MSC markers on the surface of the vesicles. As mentioned in Section 3.1, almost all MSCs expressed CD29, CD44, CD73, CD90, CD105 markers regardless of genetic modification. We found that only a small number of vesicles retained parental cell markers on their surface. Thus, the number of CD29^+^ vesicles was 23.1 ± 0.7%, 24.9 ± 1.3% and 20.5 ± 0.2% for native CIMVs, CIMVs-BFP and CIMVs-TRAIL, respectively. CD44 was detected on the surface of 72.5 ± 0.7% native CIMVs, 65.7 ± 2.1% CIMVs-BFP and 75.2 ± 1.2% CIMVs-TRAIL. Number of CD73^+^ native CIMVs, CIMVs-BFP and CIMVs-TRAIL was 50.4 ± 0.6%, 51.2 ± 1.8% and 54.8 ± 0.5%, respectively. Almost all CIMVs retained CD90 on their surface (native CIMVs, 80.4 ± 0.2%, CIMVs-BFP, 74.5 ± 1.1%, CIMVs-TRAIL, 80.9 ± 1.4%). At the same time, the number of CD105^+^ native CIMVs (22.6 ± 1.2%), CIMVs-BFP (24.5 ± 0.9%) and CIMVs-TRAIL (29.4 ± 0.7%) was significantly decreased in comparison with the parental cells (Figure 3H).

CIMVs-TRAIL were shown to contain by 6047 ± 551 times more mRNA of the *TRAIL* gene compared to native CIMVs (Figure 3I). Level of CIMVs-BFP *TRAIL* gene mRNA was also increased by 4.4 times (Figure 3I). Western blot analysis confirmed the presence of the TRAIL protein in CIMVs-TRAIL in two isoforms (28 and 34 kDa) (Figure 3J). Flow cytometry analysis of the presence of TRAIL on the membrane of the isolated vesicles showed that the protein was detected on the surface of 7.4 ± 0.9% CIMVs-TRAIL.

### 3.4. CIMVs-TRAIL Induce Apoptosis in MCF-7 Cells In Vitro

**Figure 4:**
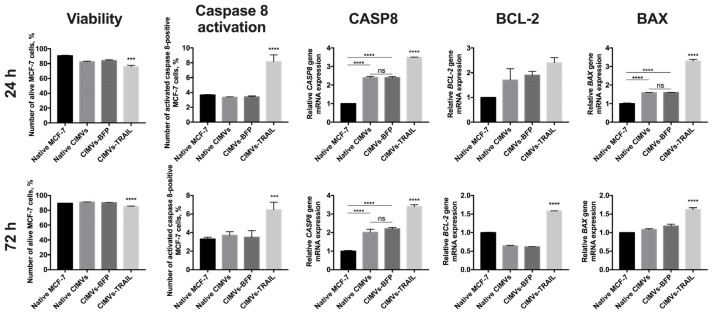
Analysis of the antitumor activity of CIMVs-TRAIL in MCF-7 tumor cell culture. The viability of tumor cells after cultivation with CIMVs was assessed using flow cytometry by staining for annexin and activated caspase 8, the level of mRNA of the apoptosis regulator genes *CASP8*, *BCL-2* and *BAX* was assessed by qPCR. The viability of MCF-7 cells was reduced by 15–20% after 24 h of cultivation with CIMVs-TRAIL. At the same time, the mRNA level of the gene of the extrinsic apoptosis pathway regulator *CASP8* and the level of activated caspase 8 protein were increased in the tumor cells cultured with CIMVs-TRAIL. The BAX/BCL2 ratio in MCF-7 cells after 24 h and 72 h of cultivation with CIMVs-TRAIL was 1.2 and 1.1, respectively, which also indicates breast cancer cells undergo apoptosis. Each box represents the mean ± SD (*n* = 3) of three replicates. **** *p*  <  0.0001, *** *p*  <  0.001, ns—not significant.

In order to analyze the antitumor activity of CIMVs-TRAIL, MCF-7 breast cancer cells were incubated with native CIMVs, CIMVs-BFP, or CIMVs-TRAIL for 24 h and 72 h, next the viability of tumor cells was analyzed by flow cytometry and the expression level of apoptosis regulator genes *CASP8*, *BCL-2* and *BAX* was determined by qPCR. According to results of annexin detection viability of MCF-7 cells was significantly reduced after 24 h of co-cultivation with CIMVs-TRAIL (75.8 ± 1.9% of alive cells) (*n* = 3, *** *p* < 0.001) compared to native CIMVs (82.4 ± 0.9% of alive cells), CIMVs-BFP (84.1 ± 1.1% of alive cells) and control tumor cells cultured without addition of the vesicles (90.7 ± 0.7% of alive cells) (Figure 4). Detection of activated caspase 8 in the tumor cells also confirmed the results. The number of alive MCF-7 cells after co-cultivation with CIMVs-TRAIL was significantly reduced (70.3 ± 0.9% of alive cells) (*n* = 3, **** *p* < 0.0001) compared to tumor cell cultured with native CIMVs (83.8 ± 0.4% of alive cells), CIMVs-BFP (84.2 ± 0.6% of alive cells) and control tumor cells cultured without addition of the vesicles (90.0 ± 0.1% of alive cells) (Figure 4). Apoptosis induction in the tumor cells was confirmed at the mRNA level by a 3.5-fold increase (*n* = 3, **** *p* < 0.0001) in the *CASP8* gene mRNA level in cells treated with CIMVs-TRAIL and a 2.4-fold increase (*n* = 3, **** *p* < 0.0001) in cells treated with native CIMVs or CIMVs-BFP compared to control without the addition of CIMVs. At the protein level the number of MCF-7 cells in which caspase 8 was activated was significantly higher in the sample cultured with CIMVs-TRAIL (8.15 ± 0.9%) (*n* = 3, **** *p* < 0.0001) compared to native CIMVs (3.35 ± 0.1%), CIMVs-BFP (3.41 ± 0.1%) and control tumor cells cultured without addition of the vesicles (3.65 ± 0.1%). Expression level of mRNA of the *BCL-2* gene, the protein of which is responsible for the suppression of apoptosis, was increased by 2.7, 1.9, and 1.7 times in CIMVs-TRAIL, CIMVs-BFP, and native CIMVs, respectively, compared to control MCF-7. While the mRNA level of the proapoptotic BAX protein gene was increased by 3.3 times (*n* = 3, **** *p* < 0.0001) in the tumor cells after cultivation with CIMVs-TRAIL, which indicates activation of the apoptotic pathway and stimulation of cell death (Figure 4). The BAX/BCL2 ratio in MCF-7 cells was 1.2 after 24 h of cultivation with CIMVs-TRAIL, which also confirms the induction of apoptosis in breast cancer cells.

After 72 h, the tumor cell viability according to the level of annexin remained reduced in the CIMV-TRAIL sample (85.0 ± 0.7% of alive cells) (*n* = 3, **** *p* < 0.0001) compared to native CIMVs (90.9 ± 0.3% of alive cells), CIMVs-BFP (90.2 ± 0.2% of alive cells) and native tumor cells (89.2 ± 0.1% of alive cells). Activated caspase 8 detection kit also confirmed the data (*n* = 3, **** *p* < 0.0001); number of alive MCF-7 after cultivation with CIMVs-TRAIL was 83.4 ± 0.9%, native CIMVs—89.7 ± 0.7%, CIMVs-BFP—90.4 ± 0.6%, native tumor cells—91.1 ± 0.6%. The induction of apoptosis was also indicated by the mRNA level of the *CASP8* gene, which was significantly increased by 3.4 times (*n* = 3, **** *p* < 0.0001) in the CIMVs-TRAIL sample compared to native MCF-7. At the same time, the mRNA level of the *CASP8* gene was also increased in tumor cell samples that were cultured with native CIMVs (by 2 times) and CIMVs-BFP (by 2.2 times) (*n* = 3, **** *p* < 0.0001), but not as significant as in the case of CIMVs-TRAIL. Analysis of activated caspase 8 protein indicated an increase only in the sample of CIMVs-TRAIL (6.46 ± 0.8%) (*n* = 3, *** *p* < 0.001), but not the rest of the cancer cells cultured with native CIMVs (3.7 ± 0.4%), CIMVs-BFP (3.5 ± 0.7%) or without treatment (3.3 ± 0.2%). The mRNA expression level of the *BCL-2* gene was 1.5-fold increased in the tumor cells after cultivation with CIMVs-TRAIL in comparison with native MCF-7, native CIMVs and CIMVs-BFP (0.6-fold increase, both) (*n* = 3, **** *p* < 0.0001). At the same time, the expression level of the *BAX* gene mRNA was changed only in the CIMV-TRAIL sample (1.6-fold increase, *n* = 3, **** *p* < 0.0001) compared to the other samples (Figure 4). The BAX/BCL2 ratio in MCF-7 cells after 72 h of cultivation with CIMVs-TRAIL was 1.1.

### 3.5. CIMVs-TRAIL Mediate Tumor Cell Death in breast Cancer Mouse Model In Vivo

**Figure 5:**
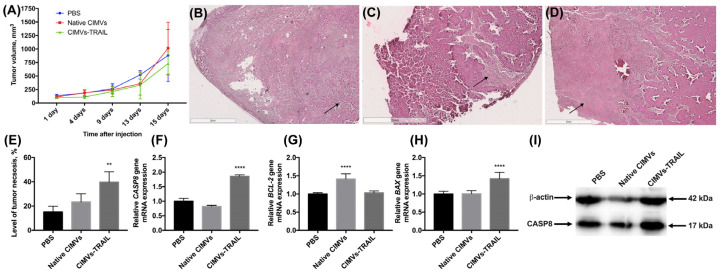
The antitumor activity of CIMVs-TRAIL was investigated in subcutaneous MCF-7 tumor xenograft mouse model. The injection of CIMVs-TRAIL did not lead to a decrease in the rate of tumor growth (**A**). However, the level of tumor tissue necrosis after injection of CIMVs-TRAIL was significantly higher (*n* = 5, ** *p* < 0.01) compared to control tumors, as seen in H&E stained tumor sections (**B**—PBS, **C**—native CIMVs; **D**—CIMVs-TRAIL), the percentage of necrotic tissue was calculated using ImageJ (**E**). At the same time, the mRNA level of the *CASP8* gene in the CIMV-TRAIL group was increased by 1.8 times (**F**), the level of the antiapoptotic *BCL-2* gene remained unchanged (**G**), and the mRNA level of the proapoptotic *BAX* gene was increased by 1.4 times (**H**). An increase in the level of a key protein involved in the process of apoptosis, CASP8, in activated form (**I**) was also observed. Each box represents the mean ± SD (*n* = 5) of five replicates. ** *p* < 0.01; **** *p* < 0.0001.

The antitumor activity of CIMVs-TRAIL was investigated using established subcutaneous MCF-7 tumor xenografts. No statistically significant difference was observed in tumor volume of treated and untreated animals (Figure 5A). However, to further assess the antitumor efficacy of CIMVs-TRAIL, after the whole course of vesicle injections tumor sections were stained with hematoxylin and eosin. It was shown that the tumors of mice injected with CIMVs-TRAIL had increased areas with eosinophilic cytosol (pink) in combination with the absence of nuclei stained with hematoxylin (blue) compared to the group that received injections of PBS or native CIMVs, indicating the necrosis. Regardless of the introduction of CIMVs, a pronounced necrotic center with tumor cells with the decreased viability was observed in the tumors. At the same time, cells replaced closer to the vascularized edge of the tumor showed high density and alive cell morphology (Figure 5B,C). However, on the sections of tumors from animals that received CIMVs-TRAIL injections, low-density areas with widely-spaced denucleated cells were observed away from the center, closer to the edge of the tumor (Figure 5D). The relative percentage of tumor tissue necrosis after injection of CIMVs-TRAIL was significantly higher (39.8 ± 8.5%, *n* = 5, ** *p* < 0.01) compared to control tumors injected with PBS (15.1 ± 4.8%) and native CIMVs (23.4 ± 6.7%) (Figure 5E). 

Further, in order to identify the pathway by which cell death was induced, an analysis of the mRNA levels of the *CASP8*, *BAX*, and *BCL-2* genes was performed. The qPCR results showed a 1.8-fold increase in the *CASP8* gene mRNA level (*n* = 5, **** *p* < 0.0001) in the group of mice injected with CIMVs-TRAIL compared to the animals injected with PBS and the native CIMVs (Figure 5F). The expression of the anti-apoptotic *BCL-2* gene mRNA in the group of animals, which received CIMVs-TRAIL, remained unchanged, while the mRNA level of the pro-apoptotic *BAX* gene was 1.4-fold increased (*n* = 5, **** *p* < 0.0001) (Figure 5G,H). The BAX/BCL-2 ratio in the tumors of the animals injected with CIMVs-TRAIL was 1.4. Interestingly, the mRNA level of the *BCL-2* gene in the tumor sample injected with native CIMVs was increased by 1.4 (*n* = 5, **** *p* < 0.0001), while the mRNA level of the *BAX* gene remained unchanged, the BAX/BCL-2 ratio was 0.7 (Figure 5).

We also analyzed the level of a key protein involved in the process of extrinsic apoptosis CASP8 in the tumor tissue. The CASP8 protein was detected in its activated isoform, and its relative level in animals injected with CIMVs-TRAIL was increased by 1.7 ± 0.2 times (*n* = 5, *** *p* < 0.001) compared to the control animals injected with PBS (1 ± 0.3) or native CIMVs (1.2 ± 0.1) (Figure 5I).

## 4. Discussion

One of the most promising therapeutic proapoptotic cytokines is TRAIL, which selectively induces apoptosis in tumors but not in healthy cells [4]. The disadvantages of recombinant TRAIL- or mAb-based therapy, such as the short half-life of the proteins, can be overcome by using cells as a TRAIL carrier in order to deliver the protein toward tumor sites. MSCs have low immunogenicity and can be easily genetically modified to express therapeutic molecules [13]. The antitumor effect of genetically modified MSCs-TRAIL has already been confirmed in various types of tumors, without any TRAIL cytotoxic effect on normal mammalian cells and tissues observed [23,24].

In our work, we genetically modified MSCs to overexpress human *TRAIL*. The resulting cell line contained 52,591 times more mRNA of the human *TRAIL* gene compared to the native MSCs. However, *TRAIL* gene expression was also slightly increased in MSCs-BFP, which is probably due to a change in the cell metabolism after genetic modification. MSCs-TRAIL also expressed TRAIL protein in two forms with a molecular weight of about 28 kDa and 34 kDa. The smaller protein is the soluble form of the TRAIL, while the 34 kDa protein represents the TRAIL monomer anchored in the membrane of cells [25]. No increase in the TRAIL protein level was observed in MSCs-BFP compared to the native MSCs. It has been shown that genetic modification and overexpression of the protein failed to induce significant changes in the viability, proliferative activity, immunophenotype, and the capability of the directed differentiation of MSCs. 

However, the overproduction of TRAIL caused significant changes in the cytokine profile of the genetically modified cells. The first group of proteins whose secretion was reduced in MSCs-TRAIL is cytokines that can support tumor cell invasion. The level of cytokines CCL21, eotaxin, CXCL1, CXCL2, CCL7, CCL19, and CCL25 was reduced in the CM of MSCs-TRAIL. CCL21 mediates intravasation mostly into different lymphatic vessels and the progression of many different types of cancer, including breast cancer [26,27,28]. Eotaxin can increase MMP-3 expression via the CCR3-ERK pathway, thereby promoting prostate cancer cell invasion and migration [29]. CXCL1 and CXCL2 are also associated with tumor progression, including tumor growth, angiogenesis, and metastasis via enhancing cell proliferation and the invasion of cancer cells [30,31]. The overexpression of CCL7 also results in the persistence of increased vascular permeability and metastasis promotion [32]. CCL19 and CCL25 can also contribute to metastasis since these cytokines induce cell migration and invasion [33,34].

The secretion of several immunomodulatory cytokines was also reduced in MSC-TRAIL CM. IL10 can promote tumor cell proliferation and metastasis via immunosuppression. Moreover, IL10 can also stimulate IL6 expression and synthesis, which can also support tumor progression, and, naturally, already oversecreted by MSCs [35]. CCL15 can also promote immune system evasion by recruiting monocytes, which in turn, become tumor-educated and accelerate tumor invasion and metastasis [36]. CCL17 is also able to modulate the tumor-supportive environment by recruiting regulatory T-cells (Tregs) and T helper 17 cells (Th17), which can suppress anti-tumor immune response [37]. CXCL5 also mediates tumor progression through neutrophil recruitment and is able to directly promote the proliferation of several types of tumor cells [38].

The most significant changes were observed in the secretion level of pro-inflammatory cytokines IL1β, IL6, and IL8. Similar to all pro-inflammatory cytokines, in the tumor microenvironment (TME), IL1 supports inflammation, which induces new-vessel formation and leukocyte recruitment as well as the formation of myeloid-derived suppressor cells (MDSCs) in the TME [39]. It has been shown that native MSCs are characterized by a consistently high secretion level of IL6 and IL8 [13]. IL6 secreted by MSCs in TME promotes cancer progression supporting increased tumor invasion, proliferation, and resistance to chemotherapy of cancer cells [40,41]. In our study, the native MSCs also expressed large amounts of IL6 (27,000–28,000 pg/mL) and IL8 (2000-11,000 pg/mL). However, genetic modification with the *TRAIL* gene led to a significant decrease in the level of these cytokines in the CM (by 4.4 and 5.3 times for IL6 and IL8 after 24 h, respectively). Probably, the overexpression of *TRAIL* leads to a change in cell metabolism, the mechanism of which requires further investigation. However, the genetic modification itself, leading to the overproduction of the protein, probably also affects the cytokine secretion profile in MSCs, since the levels of IL6 and IL8 were also slightly reduced in the MSCs-BFP sample compared to native MSCs.

The use of MSCs as a delivery vehicle for TRAIL obviously made it possible to increase the half-life of the protein and ensure the sufficient induction of the apoptosis concentration of the recombinant TRAIL in the tumor [14]. Nevertheless, a number of studies have found that systemically injected MSCs are mainly located in the lungs, liver, and spleen. Therefore, the use of vesicles from MSCs may be a more profitable strategy for the delivery of TRAIL when administered systemically since it avoids the rapid clearance and destruction of the protein and ensures a more homogeneous distribution of the vesicles throughout the body. It has been well demonstrated that TRAIL can be transported via EVs secreted by different types of parental cells that express TRAIL [20,42]. Therefore, we decided to isolate membrane vesicles from MSCs-TRAIL. Furthermore, since the yield of the exogenous membrane vesicles is comparatively low [18], the release of a high amount of vesicles from MSCs was induced using cytochalasin B. This drug inhibits actin polymerization so the CIMVs can be detached from the cell membrane by shaking [18]. The use of cytochalasin B significantly increases the yield of membrane vesicles while using low-speed centrifugation [18].

The isolated CIMVs were mostly (about 75%) 220 nm in size, which corresponds to the size of the exogeneous exosomes (200–400 nm) [17]. Other investigations of the exogenous MSC-derived vesicles also report that most parts of the EVs are 60–150 nm in size [43]. At the same time, the genetic modification and overexpression of TRAIL did not affect the size of the isolated particles, which is also confirmed by the results of other researchers [21].

We also analyzed the vesicle surface markers, as recommended by MISEV2018 [22]. CD63, CD81, and TSG101 are common markers of naturally produced EVs [44]. CD63 and CD81 are considered to be markers of the multivesicular body (MVB)-derived exosomes associated with the vesicle membrane and plasma membrane, respectively [45]. CD63 and CD81 were detected on the surface of almost all native and genetically modified CIMVs (about 70% and about 91%, respectively). At the same time, TSG101, which is also mostly detected in exosomes, was presented only in 10% of CIMVs. Similar results were observed for Hsp70 (detected in 2% of CIMVs). Both proteins, TSG101 and Hsp70, are mostly located in the cell cytoplasm [22]. Apparently, in the process of cytoplasmic membrane destruction during the release of vesicles, a small amount of the cytoplasmic contents can be captured, which does not allow the packaging of the entire repertoire of the cytoplasmic proteins of the parental cell. Additionally, calnexin was not detected, which correlates with the existing data about natural EVs since calnexin is expressed on the membrane of ER and Golgi apparatus [22].

The analysis of the presence of the surface markers of parental MSCs showed that CIMVs retain the expression profile of the CD markers of the parental cells (the number of CD90^+^ CIMVs was about 90%, CD44^+^—75%, CD73^+^—50%). At the same time, the number of CD29^+^ and CD105^+^ CIMVs was significantly reduced in comparison with MSCs, which is probably due to the fact that the density of these receptors on the cell surface is comparatively low, and they only appear on the part of CIMV population. However, the genetic modification had no effect on the number of CD markers on the surface of CIMVs. The number of cells expressing the membrane-bound form of TRAIL was about 60%. Apparently, most of the cells expressed the secreted form of the protein, which was then packaged into CIMVs. Therefore, TRAIL was detected on the surface of only a part of the vesicles.

MCF-7 are hormone-dependent breast cancer cells that are strongly resistant to TRAIL-induced apoptosis. The resistance is probably mediated by the fact that MCF7 cells only express minimal CASP8 protein and are deficient in CASP3 expression [46]. Previous studies have reported that genetically engineered MSCs with TRAIL overexpression could exhibit an inhibitory effect on the growth of some TRAIL-resistant cells, such as ileocecal adenocarcinoma HCT8 cells [47]. Moreover, iPSC-derived MSCs that were site-specifically integrated with *TRAIL* gene into the genome caused TRAIL-induced apoptosis in resistant lung adenocarcinoma A549 cells and MCF-7 cells in vitro and in vivo [48].

Additionally, several studies have been dedicated to the evaluation of the antitumor effect of natural vesicles isolated from cells overexpressing TRAIL. For example, the exosomes isolated from human leukemia K562 cells overexpressing TRAIL induced apoptosis and suppressed the growth of xenograft models of SUDHL4 lymphoma and INT12 melanoma in vivo [42]. MSC-TRAIL-derived extracellular vesicles have also shown potent antitumor activity against various types of tumor cells, including the TRAIL-resistant lung adenocarcinoma A549 cell line [21]. Our data show that CIMVs-TRAIL were also able to induce apoptosis in resistant MCF-7 cells. The induction of apoptosis was also confirmed by the ratio of the expression levels of the *BAX* and *BCL-2* genes, which was higher than 1 in tumor cells treated with CIMVs-TRAIL. A number of studies have shown a correlation between the ratio of two members of the BCL-2 protein family, BAX and BCL-2, and a sensitivity to apoptosis [49]. A BAX/BCL-2 ratio >1.00 is characteristic of apoptosis-sensitive cells and strongly correlates with up-regulated CASP3 expression and apoptosis [50]. Therefore, the change in this BAX/BCL-2 ratio in the MCF-7 cells after the addition of CIMVs-TRAIL also indicates the induction of apoptotic processes in the cells. At the same time, the mRNA level of the *CASP8* gene was 3.5 times higher, and the number MCF-7 with activated caspase 8 protein was 2.2 times higher in the cells treated with CIMVs-TRAIL, which indicates the activation of the extrinsic pathway of apoptosis, since caspase 8, as an apical caspase, provide the ligations of the death receptors [51]. Comparable data were obtained in the study of the antitumor activity of endogenous vesicles isolated from MSCs-TRAIL against resistant lung adenocarcinoma cells, where the significant apoptosis of the tumor cells was accompanied by caspase 8 activation after the addition of exosomes with TRAIL [21]. Interestingly, the induction of apoptosis and an increase in *CASP8* gene mRNA expression were also observed after the addition of native CIMVs and CIMVs-BFP, but it was significantly lower than in the CIMV-TRAIL sample. However, the increased expression of *CASP8* gene mRNA did not result in the activation of caspase 8 protein. Moreover, this induction of cell death was detected at an identical level in the samples of tumor cells cultured with native CIMVs of CIMVs-BFP. Presumably, this effect is caused by other biologically active molecules of parental MSCs carried by the vesicles. A number of studies have shown the ability of vesicles from MSCs to suppress tumor progression due to the transfer of microRNA molecules [52]. Changes in the expression level of genes involved in the intrinsic pathway of apoptosis in tumor cells after interaction with native CIMVs and CIMVs-BFP can also be mediated by microRNA carried by vesicles since it has been shown that miR-20a-3p from exogeneous MSC-derived vesicles can promote TRAIL-related apoptosis [53].

The antitumor effect of CIMVs-TRAIL was also analyzed in vivo in a xenograft breast cancer model. Multiple local administrations of CIMVs-TRAIL into the tumor were associated with the induction of tumor cell death in the CIMV-TRAIL injection group paralleled by remarkable CASP8 activation, which is generally consistent with previous studies on TRAIL antitumor activity. For example, the injection of iMSCs-TRAIL led to the induction of necrosis in tumor tissues in the animal model of breast cancer MCF-7 [48]. In addition, the level of the mRNA gene expression of the key protein of the extrinsic apoptosis pathway *CASP8* was also increased in tumor tissue injected with CIMVs-TRAIL, indicating the activation of TRAIL-mediated apoptosis in tumor cells. An increase in the level of activated caspase 8 in the tumor cells after administration of CIMVs-TRAIL was also confirmed at the protein level. The activation of the intrinsic apoptosis pathway mediated by the mitochondria was confirmed by the up-regulation of the *BAX* gene (BAX/BCL-2 ratio = 1.4).

However, despite the induction of necrosis in some areas of the tumor, the administration of CIMVs-TRAIL did not induce any significant decrease in the tumor growth rate. Apparently, this level of necrosis induction was not sufficient to influence tumor progression since, in other studies, the percentage of necrotic tissue after TRAIL delivery reached 42% [48]. Additionally, it has been shown that the introduction of vesicles together with tumor cells can more effectively delay the development of the tumor [19]. For this reason, since we started the treatment when the tumor size reached 100 mm^3^, vesicle-induced apoptosis was not enough to significantly slow tumor progression down.

In our study, the level of apoptosis induced by CIMVs-TRAIL was relatively low compared with the results obtained by other research groups analyzing the antitumor activity of MSCs-TRAIL and membrane vesicles isolated from them. For example, iMSCs-TRAIL induced apoptosis in 23% of MCF-7 cells [48], while after adding native EVs-TRAIL to A549 cells, apoptosis was detected in almost 80% of the cancer cells [21]. We assume that such a weak antitumor effect may be due to the fact that most of the TRAIL protein was located not on the surface of vesicles (only 7% of vesicles from the entire population carried TRAIL) but inside them. Additionally, although in a number of studies, MSCs expressing a secreted form of TRAIL showed a significant antitumor effect against pancreatic cancer [54] or hepatocellular carcinoma cells [55], apparently, this form of TRAIL delivery in CIMVs does not allow them to effectively bind to their receptors on the tumor cell membrane. Considering that studies in which TRAIL was located on the surface of vesicles showed the significant induction of apoptosis in vitro and inhibition of xenograft tumor growth in vivo [19], the use of the secreted form of TRAIL for packaging into vesicles is a less appropriate approach for the creation of antitumor drugs.

It should be noted that our study was carried out on a model of one type of cancer, and the antitumor effect of the obtained vesicles relative to other tumors has yet to be investigated. Moreover, the evaluation of the effectiveness of administering various CIMV-TRAIL doses and therapy duration would allow for the development of a more optimal vesicle-based treatment protocol. Another limitation of our study lies in the fact that vesicles isolated from MSCs contain a large number of biologically active molecules that can also contribute to tumor progression. This may also be the reason why the introduction of CIMVs-TRAIL had no effect on tumor growth, although it induced apoptosis in the tumor cells. Subsequent investigations aimed at studying the protein and RNA profile of the isolated vesicles will significantly expand the understanding of the biological effects of CIMVs.

## Figures and Tables

**Table 1:** Primer and probe sequences of related genes for quantitative polymerase chain reaction (qPCR).

Target Gene	Forward Primer (5′−3′)	Reverse Primer (5′−3′)	TaqMan Probe (5′−3′)
18S rRNA	GCCGCTAGAGGTGAAATTCTTG	CATTCTTGGCAAATGCTTTCG	[HEX] ACCGGCGCAAGACGGACCAG [BH2]
TRAIL	GGCACATGCCTGTAGTCCCA	GCCAGGCTGGAGTGTAGTGG	[FAM] CCACACTGCAACCTCTGCCTCCCGGG [BH1]
CASP8	CTGGACAGTGAAGATCTGGC	GATGGGTTCTTGCTTCCTTTG	[FAM] CCTGAGCCTGGACTACATTCCGC [BH1]
BCL-2	GTGGATGACTGAGTACCTGAAC	GCCAGGAGAAATCAAACAGAGG	[FAM] CAGGATAACGGAGGCTGGGATGC [BH1]
BAX	GACATGTTTTCTGACGGCAAC	AAGTCCAATGTCCAGCCC	[FAM] CTGGCAAAGTAGAAAAGGGCGACAAC [BH1]

**Table 2:** Concentration of cytokines/chemokines in the conditioned medium of native and genetically modified MSCs.

Cytokine	Concentration, pg/µL								
	24 h			48 h			72 h		
	Native MScs	MSCs-BFP	MSCs-TRAIL	Native MScs	MSCs-BFP	MSCs-TRAIL	Native MScs	MSCs-BFP	MSCs-TRAIL
**CCL21**	40.1 ± 2.1	37.7 ± 0.3	32.7 ± 0.3	44.0 ± 0.08	38.0 ± 1.3	35.2 ± 2.5	45.4 ± 0.97	43.3 ± 0.1	34.1 ± 0.4
**CXCL5**	803.7 ± 115.5	691.0 ± 16.4	418.6 ± 11.4	897.2 ± 44.7	563.6 ± 65.2	418.1 ± 12.2	547.9 ± 16.4	545.5 ± 4.8	359.9 ± 16.3
**Eotaxin1 (CCL11)**	25.5 ± 2.2	22.7 ± 0.3	18.8 ± 0.3	24.5 ± 2.0	21.1 ± 0.5	18.7 ± 0.1	29.1 ± 2.5	24.2 1.5	19.1 0.7
**Gro-** **α (CXCL1)**	1310.1 ± 1.7	640.0 ± 6.3	307.4 ± 17.7	3624.8 ± 98.0	532.2 ± 3.8	454.1 ± 3.0	885.7 ± 14.0	906.3 ± 21.0	367.1 ± 14.4
**Gro-β (CXCL2)**	18.4 ± 0.2	10.86 ± 0.09	5.4 ± 0.5	14.0 ± 0.1	7.33 ± 0.1	6.7 ± 0.5	8.0 ± 0.4	8.0 ± 0.2	6.2 ± 0.6
**IL1β**	5.8 ± 0.3	5.0 ± 0.2	3.0 ± 0.4	5.2 ± 0.2	5.3 ± 0.2	3.5 ± 0.2	9.2 ± 0.1	8.9 ± 0.5	4.0 ± 0.2
**IL6**	27346.1 ± 2595.5	24060.0 ± 783.7	6184.8 ± 98.8	12142.9 ± 1675.6	9242.1 ± 21.7	9099.9 ± 181.0	27925.7 ± 5796.7	28071.0 ± 1435.16	14239.3 ± 583.6
**IL8**	11666.5 ± 496.4	9857.5 ± 3.7	2189.0 ± 22.0	11314.3 ± 250.0	3838.4 ± 7.6	1637.6 ± 212.6	2158.4 ± 104.6	2038.0 ± 10.7	672.7 ± 19.0
**IL10**	26.7 ± 2.1	24.5 ± 1.5	17.8 ± 0.7	29.0 ± 1.0	20.2 ± 0.2	17.3 ± 0.04	18.7 ± 1.2	18.7 ± 0.2	12.0 ± 0.7
**MCP-3 (CCL7)**	30.4 ± 0.9	28.7 ± 0.4	16.23 ± 0.0	30.5 ± 0.2	25.0 ± 1.9	19.6 ± 0.5	30.7 ± 0.4	28.6 ± 0.7	22.5 ± 3.5
**MCP-4 (CCL13)**	29.8 ± 1.1	19.7 ± 0.6	12.6 ± 0.5	46.5 ± 1.7	15.7 ± 0.8	13.5 ± 0.6	19.7 ± 1.0	18.9 ± 0.4	9.4 ± 1.0
**MIP1β (CCL15)**	5.3 ± 0.1	4.4 ± 0.3	2.6 ± 0.5	5.9 ± 0.8	3.5 ± 0.3	2.8 ± 0.2	4.4 ± 0.04	4.2 ± 0.09	2.2 ± 0.5
**MIP3-β (CCL19)**	12.5 ± 1.8	10.3 ± 0.6	8.1 ± 0.6	12.6 ± 1.0	8.9 ± 0.09	7.95 ± 0.06	10.1 ± 1.1	9.0 ± 0.4	7.1 ± 0.1
**MPIF-1 (CCL23)**	7.9 ± 0.1	7.8 ± 0.8	5.4 ± 0.1	7.4 ± 0.7	7.1 ± 0.3	5.7 ± 0.8	7.8 ± 0.5	7.9 ± 0.1	5.8 ± 0.1
**CCL17**	17.6 ± 0.1	16.8 ± 0.3	13.2 ± 0.2	16.1 ± 0.4	15.8 ± 0.5	14.2 ± 0.7	18.1 ± 0.8	17.6 ± 0.7	14.3 ± 0.2
**CCL25**	93.5 ± 0.5	83.2 ± 1.7	56.9 ± 0.1	84.7 ± 2.4	75.5 ± 3.6	65.3 ± 5.7	89.4 ± 1.7	87.1 ± 1.5	67.9 ± 2.4

## Data Availability

Authors can confirm that all of the relevant data are included in the article and/or its Supplementary Materials files.

## Supplementary Materials

The following are available online at https://www.mdpi.com/article/10.3390/cimb45010038/s1, Figure S1. Immunophenotype of MSCs was analyzed using flow cytometry with the following gating strategy. Cells were positive for CD29, CD44, CD73, CD90, and CD105, and negative for (Negative control) CD11b, CD19, CD34, CD45, and HLA-DR. CD45. One of three technical replicates is shown.
