# Supplementary figures and images for: Cytochalasin B-Induced Membrane Vesicles from TRAIL-Overexpressing Mesenchymal Stem Cells Induce Extrinsic Pathway of Apoptosis in Breast Cancer Mouse Model
Source: Curr Issues Mol Biol. 2023 Jan 9;45(1):571–92. doi: 10.3390/cimb45010038 (PMC9857211; doi:10.3390/cimb45010038)

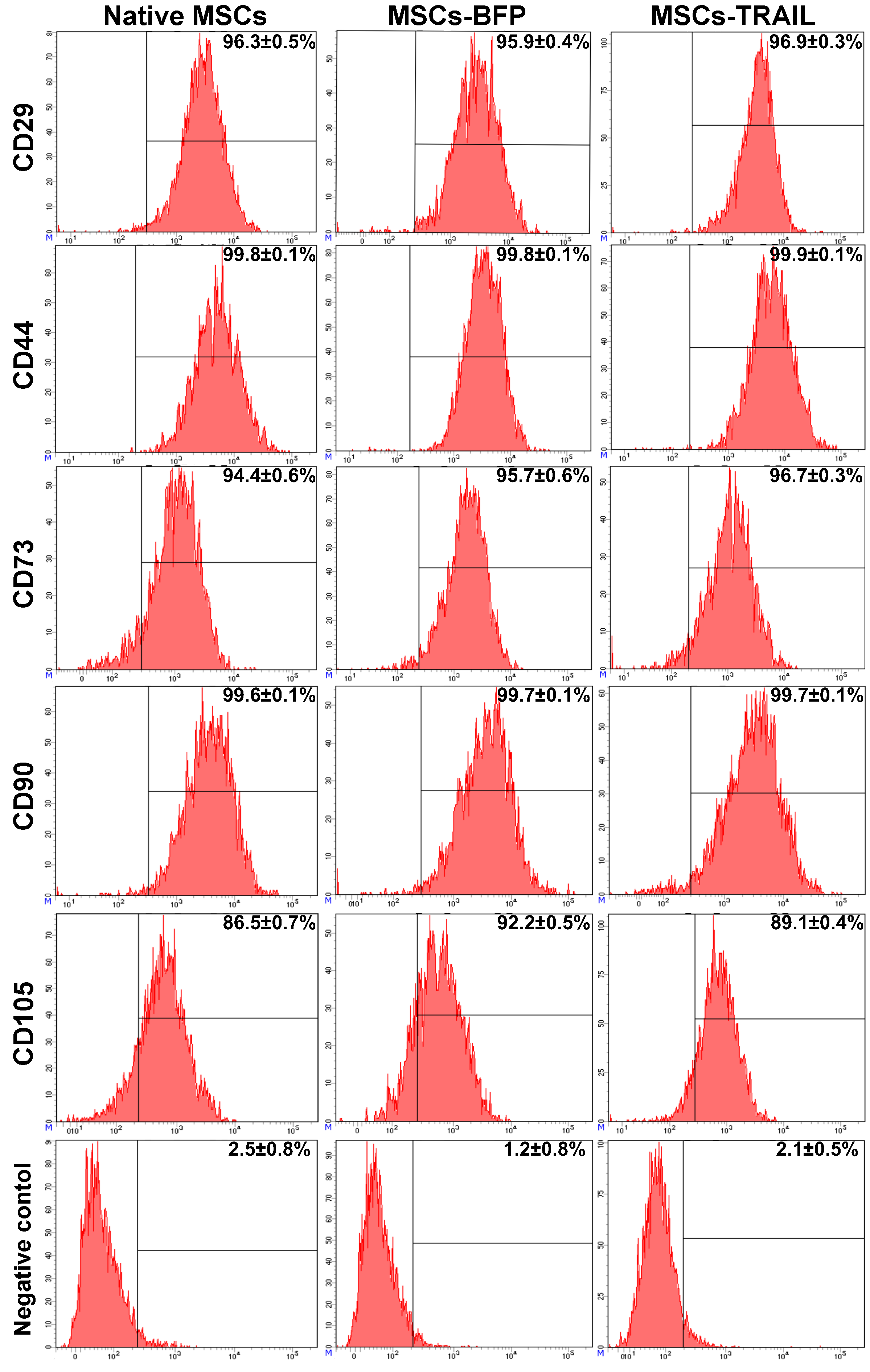

Supplement: Supplementary file 1 [file cimb-45-00038-s001.zip › Figure S1.png]
